# Supplementary material for: A temporary indwelling intravascular aphaeretic system for in vivo enrichment of circulating tumor cells
Source: Nat Commun. 2019 Apr 1;10:1478. doi: 10.1038/s41467-019-09439-9 (PMC6443676; doi:10.1038/s41467-019-09439-9)
Supplement: Supplementary file 1 — Supplementary Information [file 41467_2019_9439_MOESM1_ESM.pdf]

A temporary indwelling intravascular aphaeretic system for *in vivo* enrichment of circulating tumor cells

Kim, T. H. *et al.*

SUPPLEMENTARY INFORMATION

Figures and Tables

Supplementary Figure 1. Image of the ^{FC}GO chip and the ^{HB}GO chip during whole blood processing.

Supplementary Figure 2. Sequential image of a single MCF7 cell traveling through the ^{HB}GO chip before capture.

Supplementary Figure 3. Spatial distribution of MCF7 cells immobilized on the ^{HB}GO chip surface at varying flow rates.

Supplementary Figure 4. MCF7 capture efficiency after device sterilization at varying doses of UV exposure.

Supplementary Figure 5. Characterization of ^{HB}GO chip for CTC cluster capture

Supplementary Figure 6. Capture efficiency of ^{HB}GO chip for MCF7 cells spiked in canine blood and processed *ex vivo.*

Supplementary Figure 7. Fluorescent microscope image of human MCF7 cells and dog leukocytes stained with DAPI (blue), Cytokeratin (red), and canine CD45 (green).

Supplementary Figure 8. Number of MCF7 cells quantified from three independent experiments after intravenous injection in dogs followed by venipuncture and *ex vivo* capture using the ^{HB}GO chip.

Supplementary Figure 9. Experimental setup and quantification of CTC capture efficiency using the entire system assembly.

Supplementary Figure 10. Schematic of the integrated catheter system and its operation procedures.

Supplementary Figure 11. Schematic and operating procedure of the custom built mobile application for wireless system control and data logging.

Supplementary Figure 12. Microscopic image of the MCF7 cells after RFP transfection and DAPI staining.

Supplementary Table 1. Information of dogs and summary of MCF7 cell counts after injection. The maximum frequency of MCF7 cells identified in 1 mL of blood is shown with the time at which the sample was drawn. Accumulated cell number represents the total number of cells identified in each investigational animal after six serial blood draws.

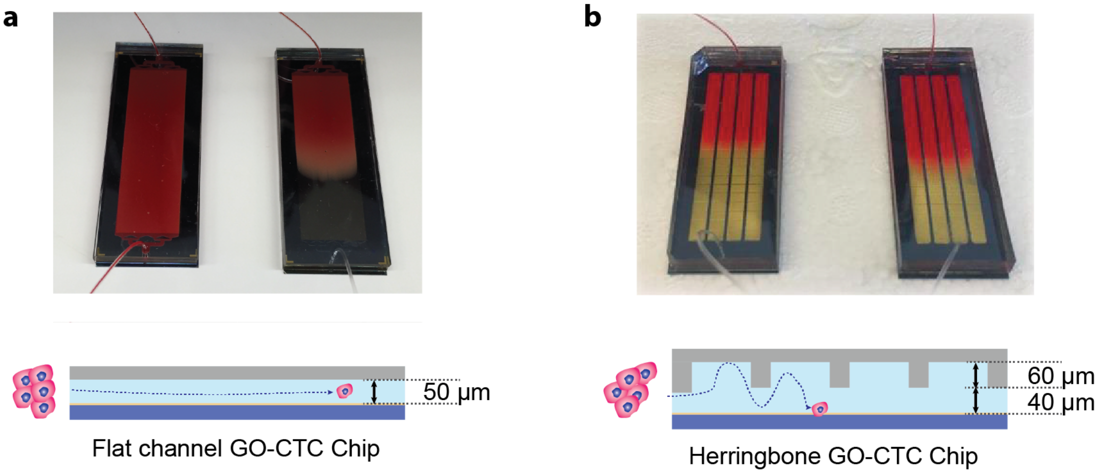

Supplementary Figure 1. Image of the ^{FC}GO chip and the ^{HB}GO chip during whole blood processing. Illustration below shows schematic of cells traveling in the two channels.

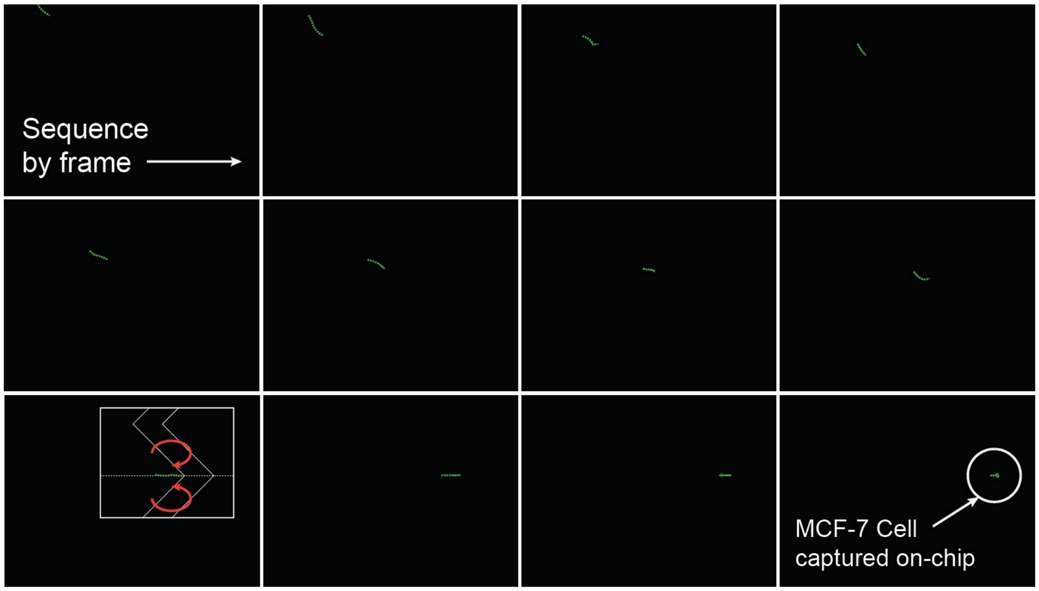

Supplementary Figure 2. Sequential image of a single MCF7 cell traveling through the ^{HB}GO chip before capture.

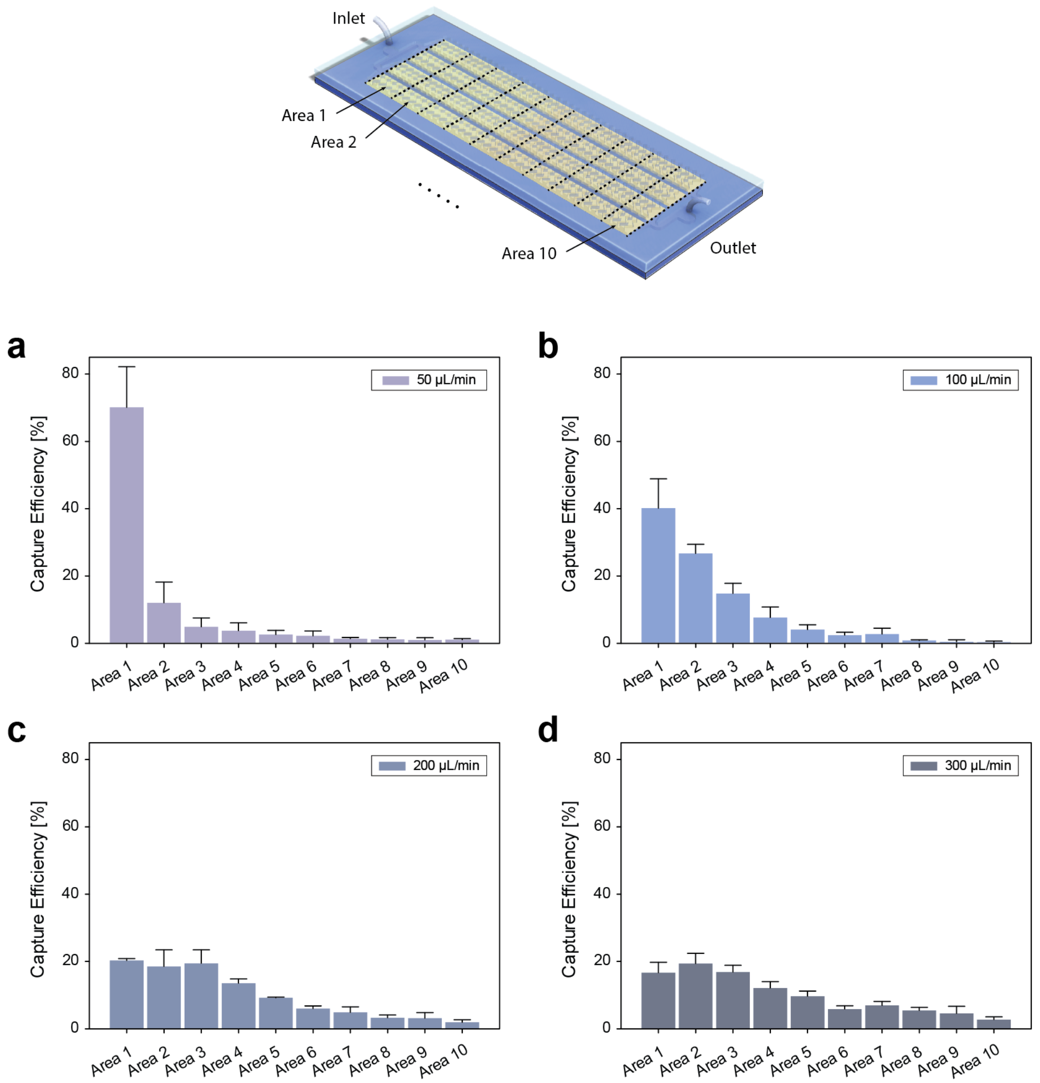

Supplementary Figure 3. Spatial distribution of MCF7 cells immobilized on the ^{HB}GO chip surface at varying flow rates: **a,** 50 μL/min; **b**, 100 μL/min; **c,** 200 μL/min; **d,** 300 μL/min. The spatial distribution was analyzed along the channel by dividing the chip surface into ten sections (data points are means ± s.d., n = 3). At a flow rate of up to 100 μL/min, more than 80% of the cells were immobilized within the first half of the ^{HB}GO chip, mostly near the inlet, suggesting the capability to efficiently isolate cells even when used in higher flow circumstances. The location of these cells became more scattered across the chip at higher flow rates reaching its maximum capacity to achieve a mean yield of > 80% at 200 μL/min.

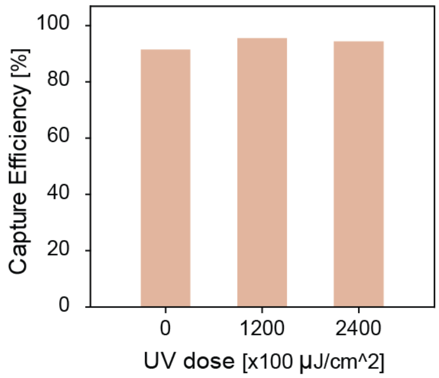

Supplementary Figure 4. MCF7 capture efficiency after device sterilization at varying doses of UV exposure.

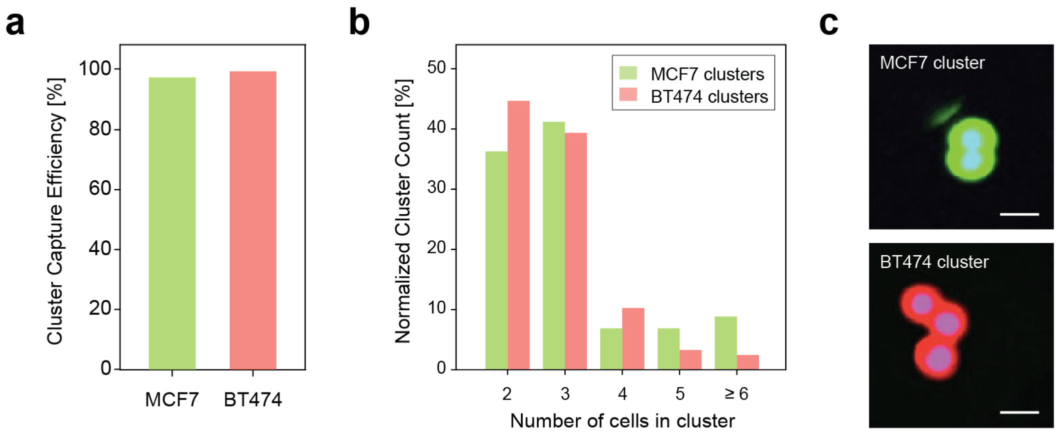

Supplementary Figure 5. Characterization of ^{HB}GO chip for CTC cluster capture. **a,** Capture efficiency of cell clusters using breast cancer cell lines. Cells were ran through a 40 µm cell strainer to form aggregates which was then placed in cell culture media to allow the clusters to enter suspension. The solution containing cell clusters was processed under an operating flow rate of 100 µL/min. **b**, Distribution of cell cluster size captured using the ^{HB}GO chip. **c,** Representative microscopic images of MCF7 and BT474 cell clusters captured on the ^{HB}GO chip surface. The majority of cell aggregates spiked into buffer to mimic CTC clusters were intact upon isolation. All scale bars represent 20 µm.

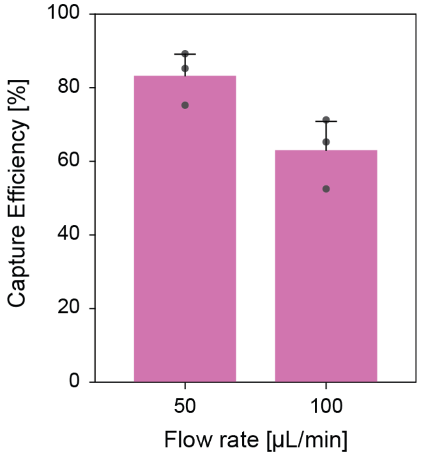

Supplementary Figure 6. Capture efficiency of ^{HB}GO chip for MCF7 cells spiked in canine blood and processed *ex vivo* (data points are means ± s.d., *n* = 3).

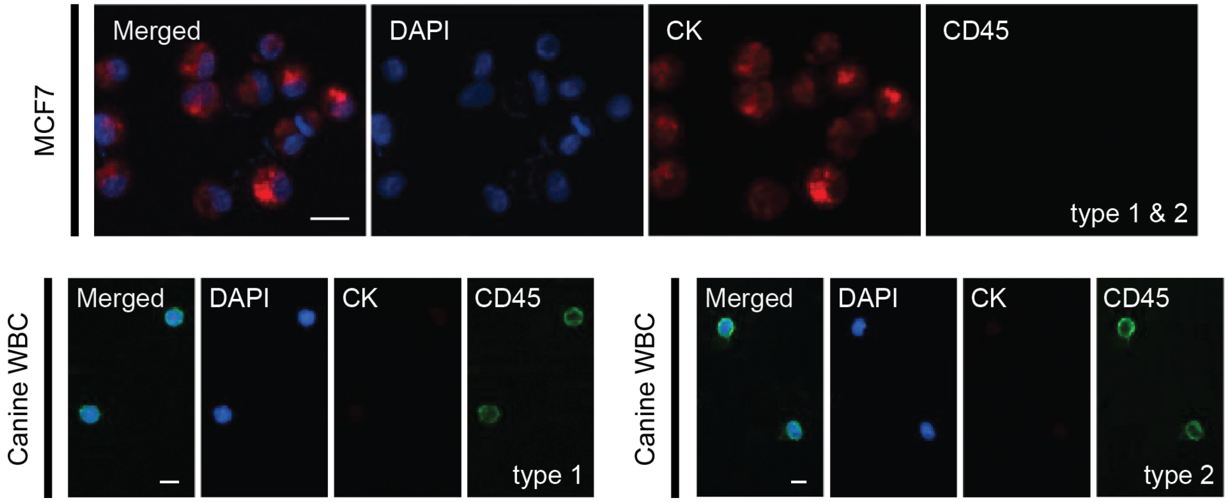

Supplementary Figure 7. Fluorescent microscope image of human MCF7 cells and dog leukocytes stained with DAPI (blue), Cytokeratin (red), and canine CD45 (green). Two anti-dog CD45 antibodies were tested (for canine WBC) and used as a cocktail (for MCF7 cell staining). All scale bars represent 20 μm.

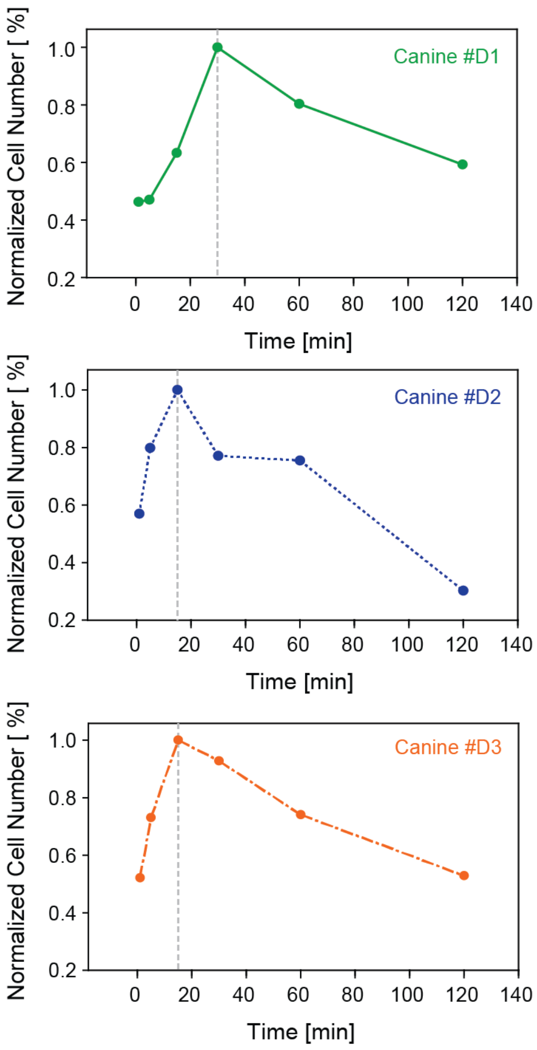

Supplementary Figure 8. Number of MCF7 cells quantified from three independent experiments after intravenous injection in dogs followed by venipuncture and *ex vivo* capture using the ^{HB}GO chip. Cell counts were normalized to the maximum frequency of MCF7 cells identified for each test.

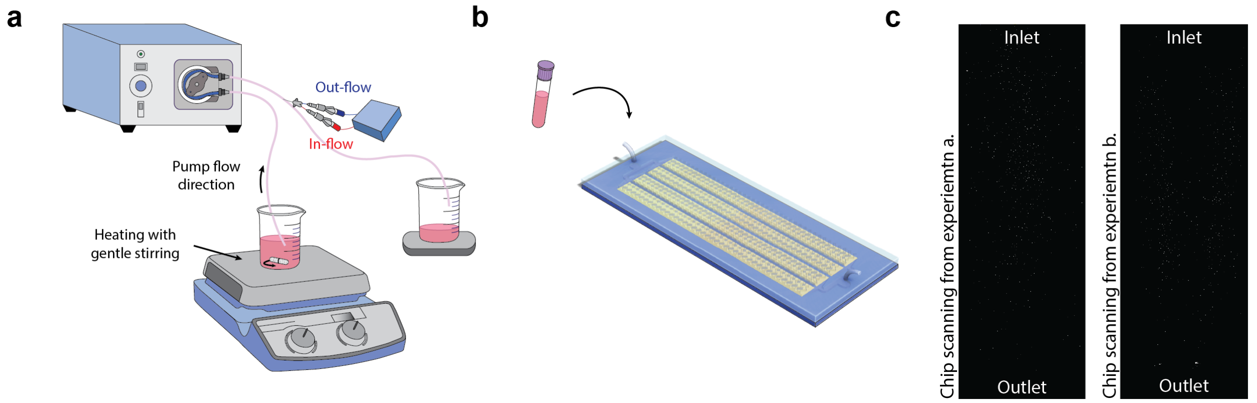

Supplementary Figure 9. Experimental setup and quantification of CTC capture efficiency using the entire system assembly. **a,** Prelabeled MCF7 cells were spiked into buffer with a concentration of 200 cells/mL and pumped through a tube (diameter of 4 mm) at a flow velocity of 1 cm/s using a peristaltic pump to mimic the blood flow with a steady-state CTC concentration. The system was connected to a catheter which was introduced into one side of the tubing and operated for 30 mins. The total the number of cells captured on the ^{HB}GO chip was 576 cells which translated into an overall capture efficiency of approximately 1 %. **b,** Similar number of MCF7 cells were also captured by processing the same volume of solution through a separate ^{HB}GO chip (543 cells), indicating that the amount of CTC capture is simply proportional to the blood processing volume from the system. This is one of the advantages of the system since CTC capture does not depend on the physiological condition (vein diameter or blood flow rate) of the human or animal subject and therefore, easier to standardize the CTC counts or the CTC concentration in blood **c,** Microscopic scanning images of the chips used in the two experiments.

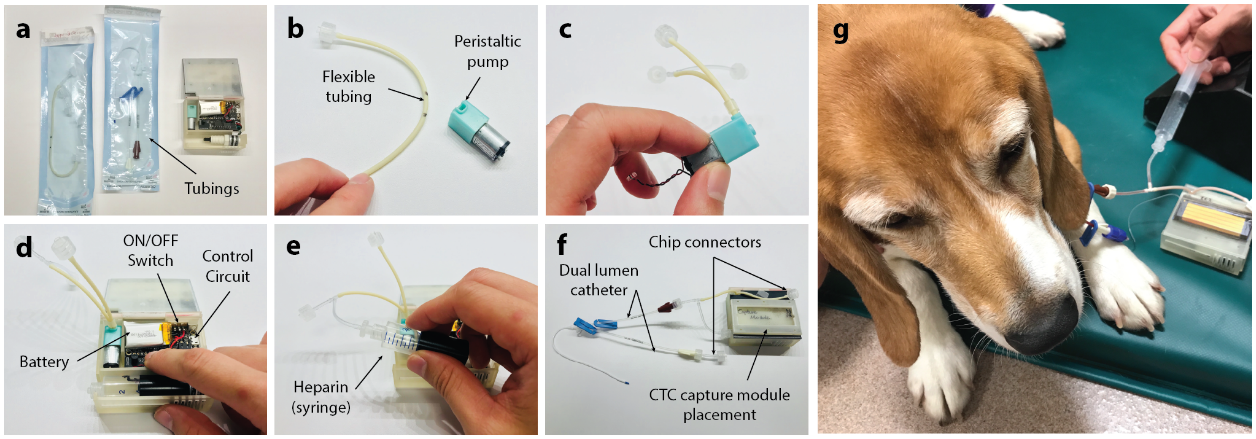

Supplementary Figure 10. Schematic of the integrated catheter system and its operation procedures. **a-c**, Connect peristaltic pump with disposable tubing units. **d-f**, Connect syringe filled with heparin, close system cover. **g**, Mount CTC capture module, prime system, connect to dual lumen catheter, and activate Bluetooth receiver to start system using wireless controller. Image shows system priming before operation.

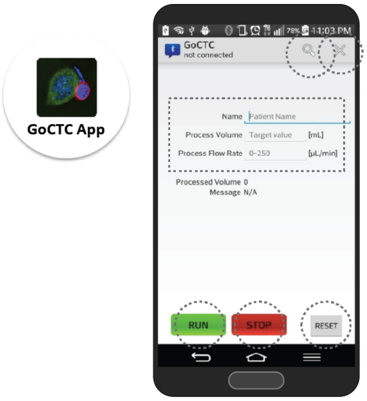

1) Open GoCTC app
2) Connect bluetooth module
3) Type in patient information
4) Set total process volume
5) Set process flow rate
6) RUN
7) STOP
8) RESET
9) Disconnect bluetooth
10) Turn off device

Supplementary Figure 11. Schematic and operating procedure of the custom-built mobile application for wireless system control and data logging.

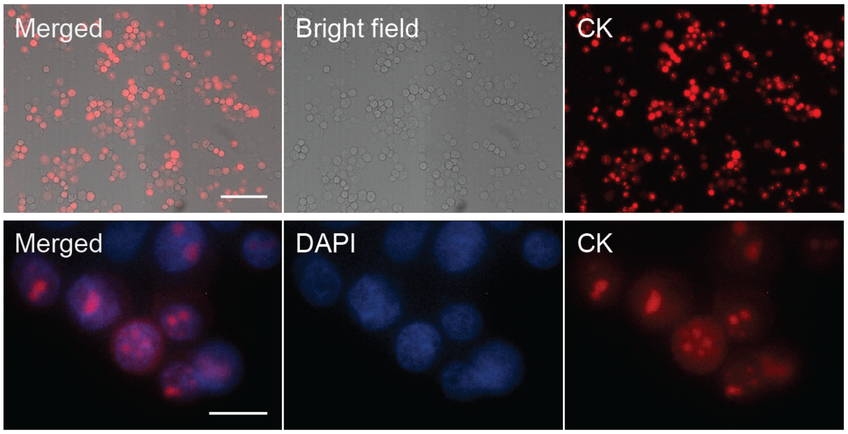

Supplementary Figure 12. Microscopic image of the MCF7 cells after RFP transfection and DAPI staining. The scale bars represent 100 (top) and 20 μm (bottom).

Supplementary Table 1. Information of dogs and summary of MCF7 cell counts after injection.

Canine ID	Type	Weight [Kg]	Max. # cells (time)	Accumulated # of cell count
D1	Beagle	12.1	90 (at 30 min)	356
D2	Beagle	13.2	122 (at 15 min)	513
D3	Beagle	13.2	134 (at 15 min)	596

* Number of MCF-7 cells Injected: 2 x 10^7 cells

The maximum frequency of MCF7 cells identified in 1 mL of blood is shown with the time at which the sample was drawn. Accumulated cell number represents the total number of cells identified in each investigational animal after six serial blood draws.
