# Supplementary material for: A temporary indwelling intravascular aphaeretic system for in vivo enrichment of circulating tumor cells
Source: Nat Commun. 2019 Apr 1;10:1478. doi: 10.1038/s41467-019-09439-9 (PMC6443676; doi:10.1038/s41467-019-09439-9)
Supplement: Supplementary file 3 — Reporting Summary [file 41467_2019_9439_MOESM3_ESM.pdf]

nature research

Corresponding author(s): Daniel F. Hayes, Sunitha Nagrath

Last updated by author(s): Feb 15, 2019

Reporting Summary

Nature Research wishes to improve the reproducibility of the work that we publish. This form provides structure for consistency and transparency in reporting. For further information on Nature Research policies, see Authors & Referees and the Editorial Policy Checklist.

Statistics

For all statistical analyses, confirm that the following items are present in the figure legend, table legend, main text, or Methods section.

n/a	Confirmed	
☐	☒	The exact sample size (*n*) for each experimental group/condition, given as a discrete number and unit of measurement
☐	☒	A statement on whether measurements were taken from distinct samples or whether the same sample was measured repeatedly
☐	☒	The statistical test(s) used AND whether they are one- or two-sided<br>*Only common tests should be described solely by name; describe more complex techniques in the Methods section.*
☒	☐	A description of all covariates tested
☒	☐	A description of any assumptions or corrections, such as tests of normality and adjustment for multiple comparisons
☐	☒	A full description of the statistical parameters including central tendency (e.g. means) or other basic estimates (e.g. regression coefficient) AND variation (e.g. standard deviation) or associated estimates of uncertainty (e.g. confidence intervals)
☐	☒	For null hypothesis testing, the test statistic (e.g. *F*, *t*, *r*) with confidence intervals, effect sizes, degrees of freedom and *P* value noted<br>*Give P values as exact values whenever suitable.*
☒	☐	For Bayesian analysis, information on the choice of priors and Markov chain Monte Carlo settings
☒	☐	For hierarchical and complex designs, identification of the appropriate level for tests and full reporting of outcomes
☒	☐	Estimates of effect sizes (e.g. Cohen's *d*, Pearson's *r*), indicating how they were calculated

Our web collection on statistics for biologists contains articles on many of the points above.

Software and code

Policy information about availability of computer code

Data collection	NIS-elements and Image J for image/video acquisition and analysis.
Data analysis	R (version 3.2.4) for two-tailed paired t-test. The mean value and standard deviation of all graphs were calculated and plotted using Sigma plot.

For manuscripts utilizing custom algorithms or software that are central to the research but not yet described in published literature, software must be made available to editors/reviewers. We strongly encourage code deposition in a community repository (e.g. GitHub). See the Nature Research guidelines for submitting code & software for further information.

Data

Policy information about availability of data

All manuscripts must include a data availability statement. This statement should provide the following information, where applicable:

- Accession codes, unique identifiers, or web links for publicly available datasets
- A list of figures that have associated raw data
- A description of any restrictions on data availability

The authors declare that all data supporting the findings of this study are available within the paper and its Supplementary Information.

Field-specific reporting

Please select the one below that is the best fit for your research. If you are not sure, read the appropriate sections before making your selection.

☒ Life sciences ☐ Behavioural & social sciences ☐ Ecological, evolutionary & environmental sciences

October 2018

Life sciences study design

All studies must disclose on these points even when the disclosure is negative.

Sample size	A minimum of 3 independent experiments were carried out for all ex vivo studies. Experiment for capturing cells, intravenously infused into the animal, was done once using the entire indwelling intravascular aphaeretic system.
Data exclusions	Data exclusion were only allowed when device failure (chip to PDMS bonding).
Replication	All the experimental findings were reliably reproduced.
Randomization	Animals were randomly chosen for each experiment.
Blinding	Data collection and analysis were not blinded.

Reporting for specific materials, systems and methods

We require information from authors about some types of materials, experimental systems and methods used in many studies. Here, indicate whether each material, system or method listed is relevant to your study. If you are not sure if a list item applies to your research, read the appropriate section before selecting a response.

Materials & experimental systems

n/a	Involved in the study	
☐	☒	Antibodies
☐	☒	Eukaryotic cell lines
☒	☐	Palaeontology
☐	☒	Animals and other organisms
☐	☒	Human research participants
☒	☐	Clinical data

Methods

n/a	Involved in the study	
☒	☐	ChIP-seq
☒	☐	Flow cytometry
☒	☐	MRI-based neuroimaging

Antibodies

Antibodies used	Anti-EpCAM antibody (BAF960, R&D Systems), anti-cytokeratin antibody (349205, BD Biosciences), anti-canine CD45 antibody (MCA1042GA and MCA2035S, Bio-Rad), secondary antibodies (A-21133, A-21121, A-11006 ,Invitrogen)
Validation	Biotinylated anti-EpCAM antibody was used for chip functionalization. Primary antibodies including anti-cytokeratin and two types of anti-canine CD45 was used with secondary antibodies conjugated with Alexa Fluor 546 or 488 to label cells (IF).

Eukaryotic cell lines

Policy information about cell lines

Cell line source(s)	MCF7 and BT474 cells were purchased from ATCC.
Authentication	Cells were authenticated by the vendor and confirmed as human by multi-species multiplex PCR
Mycoplasma contamination	Cell lines were tested for mycoplasma contamination using MycoAlertTM (Lonza) and Universal Mycoplasma Detection Kit (ATCC) and were found to be negative.
Commonly misidentified lines (See ICLAC register)	No commonly misidentified cell lines were used.

Animals and other organisms

Policy information about studies involving animals; ARRIVE guidelines recommended for reporting animal research

Laboratory animals	4 male (12-18 month old) purpose bred Beagles.
Wild animals	The study did not involve wild animals.
Field-collected samples	The study did not involve field-collected samples.

Ethics oversight	All canine experiments were performed with approval from the Colorado State University Institutional Animal Care and Use Committee (IACUC, 16-6490A)

Note that full information on the approval of the study protocol must also be provided in the manuscript.

Human research participants

Policy information about studies involving human research participants

Population characteristics	Healthy individuals (male or female) older than 18 years.
Recruitment	Voluntary
Ethics oversight	Whole blood was drawn from healthy volunteers after obtaining informed consent under an Institutional Review Board (IRB)-approved protocol at the University of Michigan.

Note that full information on the approval of the study protocol must also be provided in the manuscript.
